# Mixed emotions evoked when incongruent outward appearance and motion are combined

**DOI:** 10.3389/fnins.2026.1976417

**Published:** 2026-10-06

**Authors:** Ryousuke Kato, Mai Takafuji, Hiroshi Yamada

**Affiliations:** 1 Graduate School of Comprehensive Human Sciences, Kansei, Behavioral and Brain Sciences, University of Tsukuba, Tsukuba, Ibaraki, Japan; 2 Graduate School of Engineering, Tokyo Polytechnic University, Atsugi-shi, Kanagawa, Japan; 3 Division of Biomedical Science, Institute of Medicine, University of Tsukuba, Tsukuba, Ibaraki, Japan

**Keywords:** character, emotion, human information processing, kimo-kawaii, mixed feeling

## Abstract

Understanding mixed emotions is crucial for capturing the ambivalence of human information processing. However, how specific visual factors, such as outward appearance and motion, interact to evoke these conflicting feelings remains unclear. Here, we present three-dimensional (3D) characters as visual stimuli that evoke mixed feelings, providing empirical insights into emotional phenomena such as “kimo-kawaii” by focusing on their outward appearances and motions. Congruent stimuli (e.g., pleasant appearance with pleasant motion) and incongruent stimuli (e.g., pleasant appearance with unpleasant motion) were evaluated. Characters with congruent features evoked a single feeling: pleasant or unpleasant. In contrast, the combination of pleasant outward appearance and unpleasant motion evoked both pleasant and unpleasant feelings. Moreover, the combination of unpleasant outward appearance and pleasant motion strongly evoked unpleasant feelings rather than pleasant feelings. These findings suggest that, within the present stimulus set, outward appearance may exert a stronger influence on affective evaluation than motion. The observed co-activation of positive and negative affect may be relevant to mixed affective experiences such as “kimo-kawaii,” although further research using construct-specific measures is needed to establish this relationship.

## 1 Introduction

Emotion is a fundamental human experience. A circumplex model of affect has been proposed to describe emotional expressions within a two-dimensional circular space: valence (i.e., positive–negative) and arousal (i.e., high–low) (Russell, 1978). Emotional responses have been induced using emotional photographs in many previous studies, such as those using the international affective picture system (IAPS) (Lang et al., 2008), movies (Larsen et al., 2001), colors (Omori et al., 2002), and stimulus patterns (Cole and Wilkins, 2013). Previous studies have examined how different types of affective information contribute to emotional evaluation. For example, mixed emotions have been studied using situations or stimuli that simultaneously provide positive and negative emotional information, and a meta-analysis demonstrated that mixed emotions can be elicited across different types of emotional induction and measurement paradigms (Larsen et al., 2001; Berrios et al., 2015). In research involving characters and virtual agents, both outward appearance and dynamic behavior have also been shown to influence affective impressions (Sugano et al., 2015; Nonomura et al., 2015; Numata et al., 2020). Furthermore, previous work has demonstrated that characteristics of facial motion can influence the perception of virtual characters and their perceived uncanniness (Tinwell et al., 2011). These findings indicate that different visual characteristics can contribute to affective evaluation and that conflicting or incongruent information may alter emotional impressions.

However, it remains unclear how incongruency between static outward appearance and dynamic motion within the same character contributes to the simultaneous experience of positive and negative affect. In particular, previous studies have generally examined individual visual characteristics or relationships between emotional information and behavior, whereas the present study independently manipulates the emotional valence of outward appearance and motion within the same 3D character. This approach allows us to examine whether a specific combination of affectively incongruent visual characteristics is associated with co-activation of positive and negative affect.

Both primitive (e.g., anger, happiness, and fear) and complex emotions (e.g., mixed feelings) have been studied recently. The concept of mixed feelings underlies the assumption that human emotions are mixtures of multiple primitive emotions, including conflicting emotions. A particular situation or visual stimulus may evoke positive and negative emotions simultaneously. For example, mixed feelings are common experiences at graduations, as these events are both celebrations and farewells (Larsen et al., 2001). Namely, both happy emotions due to the celebratory situation and sad emotions due to the parting situation are evoked simultaneously. In this example, these mixed feelings are based on memories.

Other mixed feelings are based not on memory, but on the visual impression of stimuli. About 20 years ago, a new slang word has emerged among young people to express mixed feelings based on impression in Japan: “kimo-kawaii (gross cute)” (Miller, 2011; Bonnah, 2019), which had emerged as a new word around 2006 (Ihara and Nittono, 2024). Visual stimuli evaluated as “kimo-kawaii” elicit both pleasant emotions [e.g., cute or kawaii (Bonnah, 2019; Nittono, 2011; Laohakangvalvit et al., 2017; Marcus et al., 2017; Takada, 2016)] and unpleasant emotions [e.g., eerie or disgusting (Marcus et al., 2017; Takada, 2016)]. Furthermore, previous studies focusing on “kimo-kawaii” from a cultural perspective suggest that the preference for such characters is deeply linked to social suppression unique to Japan (Bonnah, 2019). An example of a mascot to illustrate “kimo-kawaii” is Sento-kun, which is used in Nara City’s public relations activities (Akizuki, 2010). Sento-kun looks like a young Buddhist priest, but the deer antlers on his head evoke feelings of creepiness because deer antlers are incongruent with the head of a young man. The term “kimo-kawaii” is often used in relation to character-like or anthropomorphic entities, including mascots and other living-like stimuli (Bonnah, 2019; Akizuki, 2010). However, its conceptual boundaries and the extent to which the associated mixed affect generalizes across different stimulus categories remain unclear. These types of mixed feelings based on incongruent factors of the character are likely to be evaluated momentarily, in contrast to mixed feelings based on experiences (e.g., those evoked on graduation day) (Larsen et al., 2001).

The psychological underpinnings of these feelings, as delineated in previous studies (Marcus et al., 2017; Takada, 2016), exhibited slight variations. For instance, one study (Marcus et al., 2017) defined “kimo-kawaii” as the combination of the concepts of “kawaii” and “eerie.” Another study (Takada, 2016) defined “kimo-kawaii” as consisting of “kawaii” and “disgusting.” The two definitions under consideration include the concept of “kawaii,” which can be translated as “cute” or “pleasing.” However, the ‘kimo-’ element, corresponding to unpleasant emotions, differed slightly, and the shared understanding is not yet fully established. In addition, it is experimentally unclear what role the incongruent factor plays in evoking “kimo-kawaii” mixed emotions.

To examine “kimo-kawaii” mixed feeling, we assume “kimo-kawaii” as a mixed emotion consisting of emotions with opposite valence, because “kimo-kawaii” mixed emotion could be evoked when both pleasant emotion and unpleasant emotion are evoked at the same time. In the present study, we examined a single emotional dimension (i.e., valence) as emotional factor and also used outward appearance stimuli and motion stimuli. We created separate sets of stimuli with outward appearances and motions eliciting different emotional valences, which could elicit incongruent emotional responses. Using these combined stimuli, we examined the emotions when incongruent outward appearance and motion were combined. If the “kimo-kawaii” mixed feeling is evoked by viewing incongruent stimuli, emotions of opposite valence (i.e., pleasant emotion and unpleasant emotion) will be evoked simultaneously.

## 2 Methods

Sixteen university students aged 22–33 years (mean age = 25.7 years, SD = 4.11, 9 male and 7 female) participated in the main study. The participants in the main study did not participate in the pilot study (mean age = 22.0 years, SD = 1.46, 12 male and 4 female). Written informed consent was obtained from all participants prior to the experiment. This study was approved by the ethics committee of the University of Tsukuba (approval number: 26-114).

### 2.1 Study design

We used human-like three-dimensional (3D) characters as the visual stimuli. We selected human-like 3D characters to maintain a consistent character context while independently manipulating outward appearance and motion. In the pilot study, we created 10 3D characters for different outward appearance without motion, i.e., outward-appearance effect only. They were created assuming that half of them were pleasant and the other half were unpleasant. In addition, 10 motions were applied to neutral outward appearance 3D characters, i.e., motion effect only. These twenty 3D characters were prepared, assuming that half of them were pleasant and the other half were unpleasant in outward appearance and motion effect, respectively. Each of twenty 3D characters was evaluated as pleasant or unpleasant by the participants, and we used the top two pleasant or unpleasant outward appearance and motion for the main study, i.e., four outward appearance stimuli and four motion stimuli, respectively. In the main study, the selected 3D characters and motions were combined, i.e., sixteen 3D characters. These combined stimuli (e.g., pleasant 3D characters with unpleasant motion and vice versa) were evaluated, i.e., mixed effect of outward appearance and motion in 3D visual stimuli. This procedure allowed us to examine whether incongruent visual stimuli evoke simultaneous pleasant and unpleasant affective responses that may be relevant to “kimo-kawaii.” This extreme-stimulus selection strategy was implemented as a proof-of-concept approach. By pairing the most contrasting pleasant and unpleasant features, we established clear stimulus boundaries to effectively isolate and determine whether structural incongruity between outward appearance and motion can evoke mixed feelings.

To examine the specific effect of the combination, we compared the evaluation score of the pilot study to that of the main study using the different participants from those in the pilot study. This procedure allowed us to determine whether a character including opposite emotional features evokes simultaneous pleasant and unpleasant affective responses when participants experienced the stimuli for the first time. We examined whether the evaluation scores reflected the interaction effect of the opposite emotions or the individual effects of each emotion. If the individual factor evoked each emotion, the mean score of outward appearance and motion in the pilot study will be same as that of the combination of the outward appearance and motion. On the other hand, if the interaction is observed, the evaluation score of the main study will be different from that of the pilot study.

### 2.2 Stimuli used in the pilot and main studies

Twenty visual stimuli were presented (Figure 1) in the pilot study. Half of the visual stimuli were unique 3D characters (outward appearance stimuli), and the other half were motion stimuli only using neutral outward appearance character. All stimuli were created using 3D computer graphics software (Blender, ver.2.60). All outward appearance stimuli were positioned in a T-pose as the neutral pose and displayed in front, back, right, and left views. All motion stimuli were displayed using an emotionally neutral outward appearance 3D character for approximately 10 s. Examples of all stimuli are presented in the URL as part of the Appendix.

**Figure 1:**
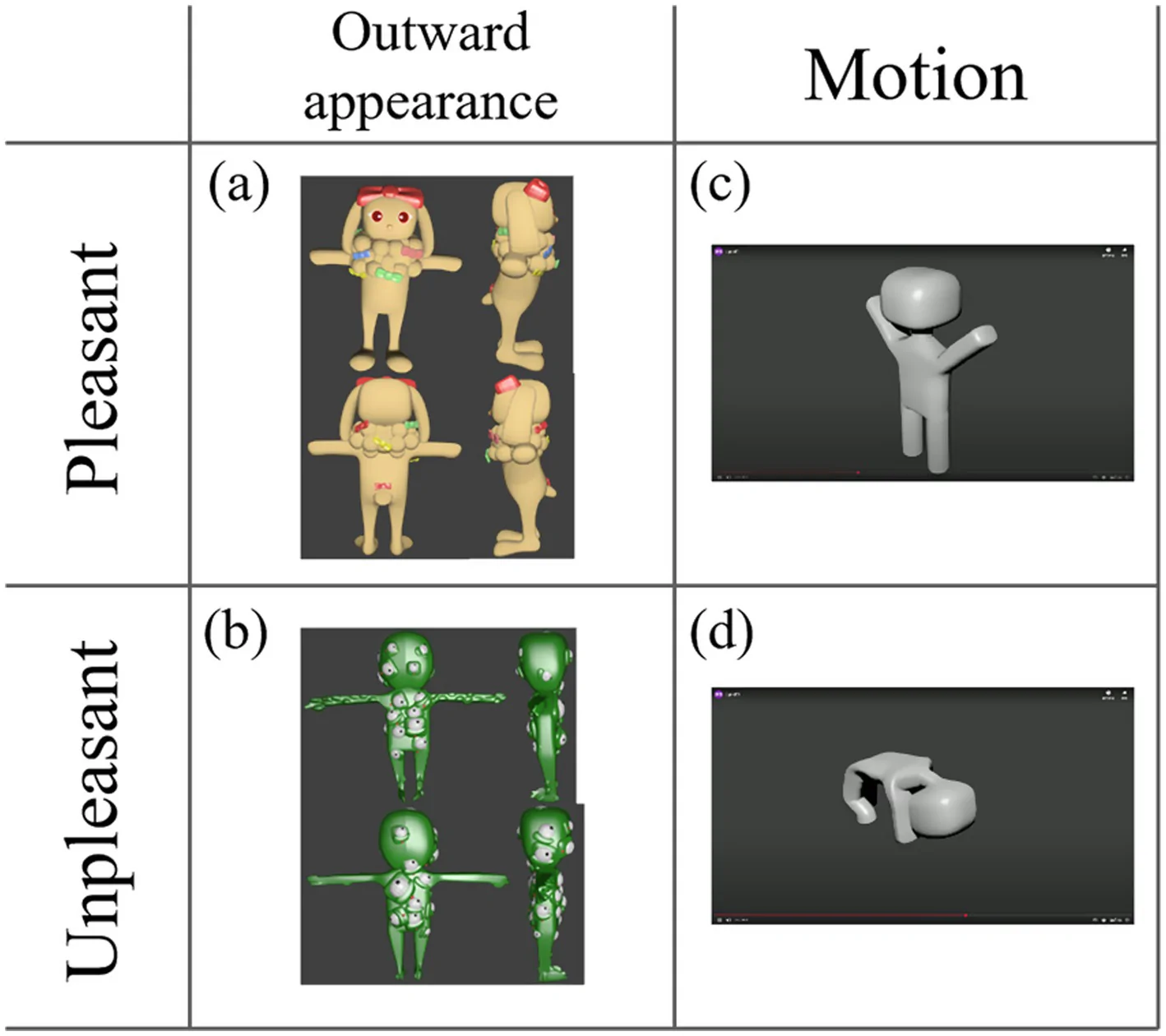
Examples of stimuli used in the pilot study. Twenty stimuli were prepared for the pilot study. See the Methods section for details. **(a)** Pleasant outward appearance (No. 2 in Supplementary Table 1). **(b)** Unpleasant outward appearance (No. 10 in Supplementary Table 1). **(c)** Pleasant motion (No. 11 in Supplementary Table 1). **(d)** Unpleasant motion (No. 16 in Supplementary Table 1).

In the pilot study, the participants were required to view the outward appearance and motion stimuli, while they evaluated each stimulus using a questionnaire. The order of the presented stimuli was randomized for each participant. The outward appearance stimuli were presented until the participants began the evaluation. The motion stimuli were presented for approximately 10 s until the participants began the evaluation.

Twenty-four evaluation items from a multiple mood scale (Terasaki et al., 1992) were chosen from the 80-item group and used for the evaluation of stimuli in the pilot study (Table 1). These items comprised 9 pleasant items, 9 unpleasant items, and 6 filler items. For pleasant items, 3 items were randomly chosen from each of the “actively pleasure” group, “inactively pleasure” group, and “attachment” group. For unpleasant items, 3 items were randomly chosen from each of the “depression and anxiety” group, “hostile feelings” group, and “fatigue” group. For filler items, 6 items were randomly chosen from each of the “surprising” group and “concentration” group. The participants were required to evaluate all stimuli using a 5-point scale (1 = do not feel, 5 = strongly feel) for each of the 24 evaluation items. The order of the 24 items in the evaluation questionnaires was randomized.

**Table 1:** All items used in the pilot and main studies.

Japanese	English
Pleasant items	
はつらつとした	Energetic
活気のある	Lively
陽気な	Cheerful
ゆったりとした	Relaxed
のんびりした	Taking it easy
やわらいだ	Calm
かわいらしい	Lovely/cute
かれんな	Charming/pretty
いとおしい	Sweet
Unpleasant items	
無関心な	Apathetic
無気力な	Drained
疲れた	Tired
悩んでいる	Worried
くよくよした	Childishly regretful
不安な	Uneasy
気分を害した	Unpleasant
むっとした	Offended
憎らしい	Hateful
Filler items	
はっとした	Startled
驚いた	Surprised
ぼうぜんとした	Shocked
慎重な	Careful
緊張した	Tense
用心深い	Cautious

Stimuli were evaluated using the Japanese terms shown on the left. Corresponding English translations are provided on the right.

The pleasantness and unpleasantness scores acquired in the evaluation were averaged for each stimulus. We analyzed the data from 15 participants (4 female, 11 male) for outward appearance, because one male participant did not evaluate part of the outward appearance stimuli. Mean pleasantness and unpleasantness evaluation scores as well as the differences between each set of evaluation scores are shown in Table 2. These scores were acquired from the questionnaire in the pilot study. Based on these differences, we created four stimuli with pleasant outward appearances and unpleasant motions by combining the two stimuli with the most pleasant outward appearances (Nos. 2 and 4 in Table 2) and two stimuli with the most unpleasant motions (Nos. 16 and 18 in Table 2). These stimuli correspond to Nos. 5–8 in Supplementary Table 2. Likewise, we created four other stimuli with unpleasant outward appearances and pleasant motions by combining the two stimuli with the most unpleasant outward appearances (Nos. 7 and 10 in Table 2) and two stimuli with the most pleasant motions (Nos. 11 and 12 in Table 2). These stimuli correspond to Nos. 9–12 in Supplementary Table 2. The other eight stimuli were congruent stimuli constructed from the stimuli with the most extremely evaluations (i.e., pleasant stimuli: Nos. 2, 4, 11, and 12; unpleasant stimuli: Nos. 7, 10, 16, and 18 in Table 2). We created 16 stimuli in all for this study (Figure 2).

**Table 2:** Mean evaluation scores (SD) for each stimulus and their differences in the pilot study.

Type of stimuli	Outward appearance evaluation score			Differences	*t*-test	Motion evaluation score			Differences	*t*-test
	ID of stimuli	Pleasantness	Unpleasantness			ID of stimuli	Pleasantness	Unpleasantness		
Pleasant stimuli	4	3.12 (0.60)	1.41 (0.17)	**1.71**	*p* < 0.001	12	3.15 (0.70)	1.42 (0.22)	**1.73**	*p* < 0.001
	2	3.28 (0.30)	1.68 (0.23)	**1.6**	*p* < 0.001	11	3.08 (0.40)	1.60 (0.34)	**1.47**	*p* < 0.001
	3	3.04 (0.49)	1.64 (0.17)	1.39	*p* < 0.001	14	2.63 (0.55)	2.01 (0.61)	0.62	*p* < 0.05
	1	3.10 (0.46)	1.72 (0.20)	1.38	*p* < 0.001	15	2.38 (0.33)	1.88 (0.23)	0.49	*p* = 0.003
	5	2.78 (0.32)	1.92 (0.34)	0.86	*p* < 0.001	13	2.27 (0.67)	2.86 (0.86)	−0.6	*n.s.*
Unpleasant stimuli	8	1.68 (0.27)	2.38 (0.47)	−0.69	*p* = 0.002	17	2.96 (0.86)	1.59 (0.29)	1.37	*p* < 0.001
	9	1.76 (0.42)	2.73 (0.46)	−0.97	*p* < 0.001	20	2.56 (1.21)	1.64 (0.39)	0.92	*n.s.*
	6	1.39 (0.43)	2.75 (0.66)	−1.36	*p* < 0.001	19	1.78 (0.62)	2.21 (0.47)	−0.43	*n.s.*
	10	1.26 (0.23)	2.78 (0.60)	**−1.52**	*p* < 0.001	18	1.99 (0.49)	2.45 (0.73)	**−0.46**	*n.s.*
	7	1.46 (0.23)	3.52 (0.50)	**−2.06**	*p* < 0.001	16	1.58 (0.41)	2.74 (0.68)	**−1.16**	*p* < 0.001

The bold values indicate the stimuli selected from the pilot study for use in the main study.

**Figure 2:**
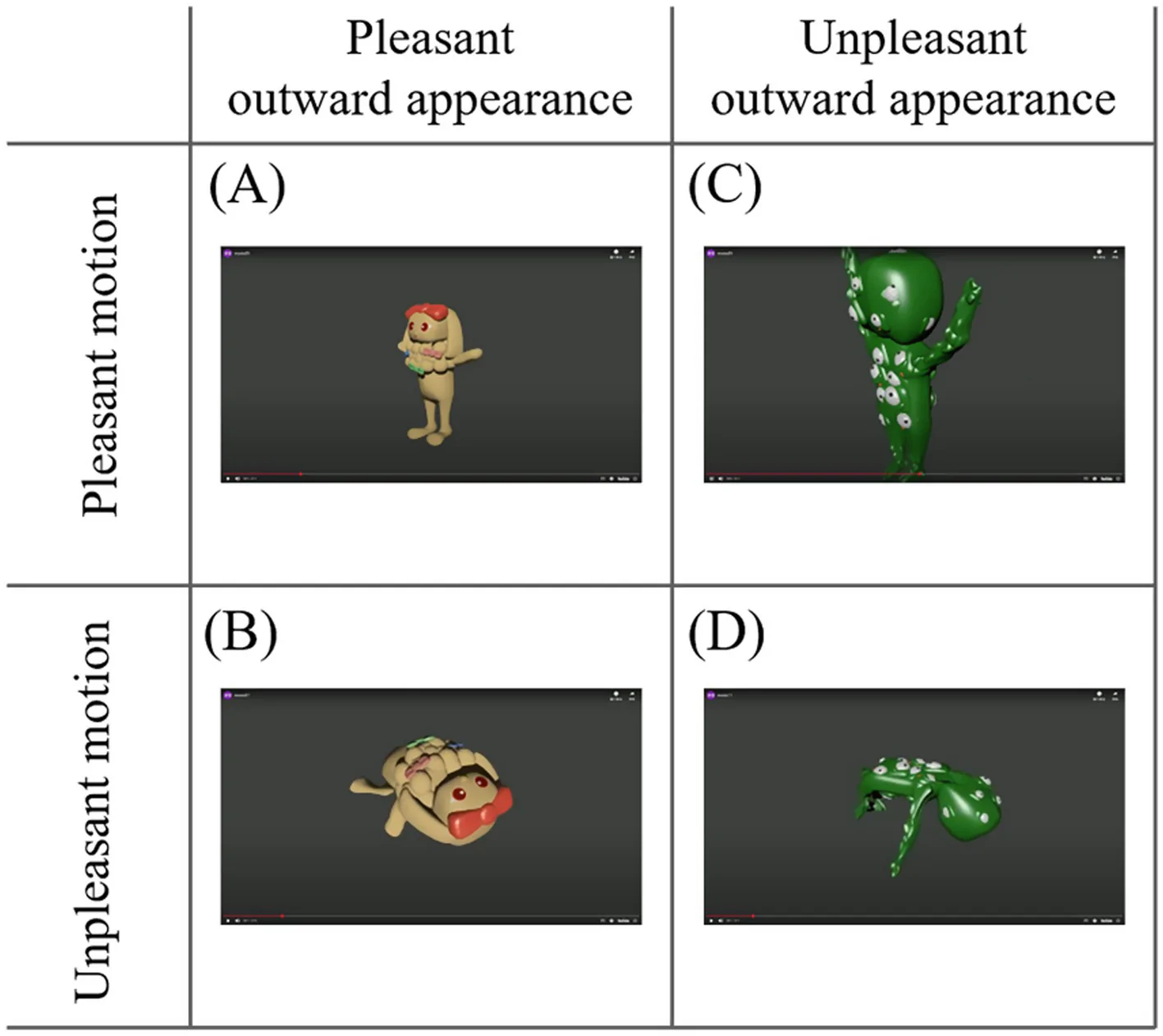
Examples of stimuli used in the main study. Sixteen stimuli were prepared by combining outward appearance and motion. See the Methods section for details. **(A)** Pleasant appearance and pleasant motion (No. 3 in Supplementary Table 2). **(B)** Pleasant appearance and unpleasant motion (No. 7 in Supplementary Table 2). **(C)** Unpleasant appearance and pleasant motion (No. 9 in Supplementary Table 2). **(D)** Unpleasant appearance and unpleasant motion (No. 13 in Supplementary Table 2).

### 2.3 Procedure in the main study

Sixteen 3D characters (i.e., four outward appearances × four motion stimuli) were used as the visual stimuli (Supplementary Table 2). All stimuli were presented for approximately 10 s. The questionnaire used in the pilot study was also used in the main study. Other procedures were the same as those described for the pilot study.

### 2.4 Data analysis

The pleasantness and unpleasantness scores acquired in the evaluation were averaged for each combination group. Two-way analyses of variance (ANOVAs) for outward appearance (pleasantness or unpleasantness) and motion (pleasantness or unpleasantness) were performed to examine the effects of each factor on the pleasantness and unpleasantness evaluation scores separately.

To examine the effect of combination in the main study, *t*-tests (the pilot study vs. the main study) were performed for each of the four types of combination stimuli separately for pleasantness score and unpleasantness score. For the pilot study, the mean scores corresponding to the combination of the main study were examined. For example, if pleasant outward appearance and unpleasant motion were combined in the main study, the mean scores of pleasant outward appearances (Nos. 2 and 4 in Table 2) and unpleasant motions (Nos. 16 and 18 in Table 2) in the pilot study were averaged. This mean score of the pilot study was compared to the mean score of the main study.

A sensitivity power analysis was conducted using G*Power 3.1.9.7. For the 2 × 2 within-subjects interaction, which has one degree of freedom, the interaction can be tested equivalently using a difference-in-differences contrast. Accordingly, a paired-samples t-test framework was used for the sensitivity analysis. Given the sample size of 16 participants, *α* = 0.05, and desired power of 0.80, the minimum detectable effect size for the interaction effect in the within-subjects design was dz. = 0.75. The observed partial eta-squared values for the primary main effects and interaction effects ranged from *η_p_*^2^ = 0.347 to 0.834. Although the observed effects were large in magnitude, the sensitivity analysis indicates that the present sample size had limited sensitivity to smaller effects. Therefore, these findings should be interpreted with caution.

## 3 Results

The mean pleasantness and unpleasantness evaluation scores acquired from the questionnaire are shown in Figure 3 and Table 3. These scores were analyzed by using a Two-way repeated-measures ANOVA, performed on each evaluation score, with outward appearance (2) and motion (2) as within-subject factors. The results are detailed below.

**Figure 3:**
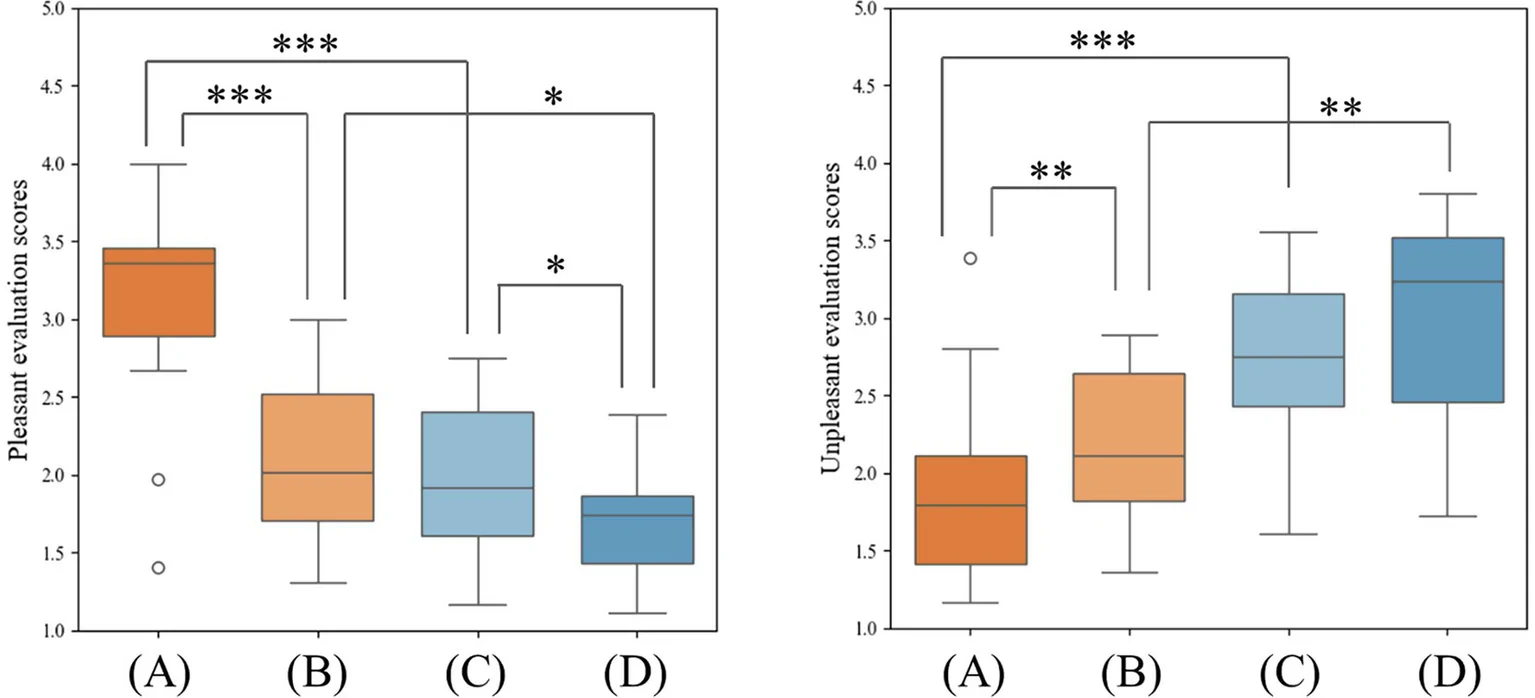
Pleasantness and unpleasantness evaluation scores in the main study. Box plots show the scores for each condition. The line within each box indicates the median, and the lower and upper edges indicate the first and third quartiles. Whiskers extend to the most extreme values within 1.5 times the interquartile range; points beyond the whiskers indicate outliers. Left: pleasantness scores. Right: unpleasantness scores. **(A)** Pleasant appearance and pleasant motion; **(B)** pleasant appearance and unpleasant motion; **(C)** unpleasant appearance and pleasant motion; **(D)**. unpleasant appearance and unpleasant motion. Brackets and asterisks indicate significant differences between conditions after Holm–Bonferroni correction (*p < 0.05, **p < 0.01 ***p < 0.0001).

**Table 3:** Mean evaluation scores (SD) in the main study.

Evaluation score	Condition	Congruent/Incongruent	Outward appearance	Motion	Score
Pleasantness score	(A)	Congruent	Pleasant	Pleasant	3.11 (0.64)
	(B)	Incongruent	Pleasant	Unpleasant	2.08 (0.49)
	(C)	Incongruent	Unpleasant	Pleasant	1.98 (0.50)
	(D)	Congruent	Unpleasant	Unpleasant	1.69 (0.37)
Unpleasantness score	(A)	Congruent	Pleasant	Pleasant	1.67 (0.37)
	(B)	Incongruent	Pleasant	Unpleasant	2.43 (0.45)
	(C)	Incongruent	Unpleasant	Pleasant	2.71 (0.54)
	(D)	Congruent	Unpleasant	Unpleasant	3.01 (0.66)

### 3.1 Pleasantness evaluation scores in the main study

We first examined how positive feelings were evoked by the outward appearance and motion in the main study (Figure 3, left). The main effect of outward appearance was significant, indicating that pleasant appearance (Conditions A and B) evoked significantly more positive feelings than unpleasant appearance (Conditions C and D) (*F* (1, 15) = 53.6, *p* < 0.0001, *η_p_*^2^ = 0.781). Similarly, we found a significant main effect of motion, with pleasant motion (Conditions A and C) evoking more positive feelings than unpleasant motion (Conditions B and D) (*F* (1, 15) = 75.4, *p* < 0.0001, *η_p_*^2^ = 0.834). These observations were consistent with the evaluation scores in the pilot study, which examined different participants (Tables 3–5; see the last section for more details).

**Table 4:** Mean evaluation scores (SD) in the pilot study.

Evaluation score	Condition	Outward appearance	Motion	Score
Pleasantness score	(a)	Pleasant	Neutral	3.20 (0.88)
	(b)	Unpleasant	Neutral	1.36 (0.35)
	(c)	Neutral	Pleasant	3.07 (0.74)
	(d)	Neutral	Unpleasant	1.77 (0.76)
Unpleasantness score	(a)	Pleasant	Neutral	1.54 (0.55)
	(b)	Unpleasant	Neutral	3.15 (0.74)
	(c)	Neutral	Pleasant	1.53 (0.52)
	(d)	Neutral	Unpleasant	2.65 (0.86)

**Table 5:** Mean combined evaluation scores (SD) from the pilot study for comparison with the main study.

Evaluation score	The compared groups in the main study	Outward appearance in the pilot study	Motion in the pilot study	Combined score of the pilot study
Pleasantness score	(A)	Pleasant (a)	Pleasant (c)	3.14 (0.66)
	(B)	Pleasant (a)	Unpleasant (d)	2.49 (0.58)
	(C)	Unpleasant (b)	Pleasant (c)	2.21 (0.39)
	(D)	Unpleasant (b)	Unpleasant (d)	1.56 (0.37)
Unpleasantness score	(A)	Pleasant (a)	Pleasant (c)	1.54 (0.40)
	(B)	Pleasant (a)	Unpleasant (d)	2.10 (0.51)
	(C)	Unpleasant (b)	Pleasant (c)	2.34 (0.44)
	(D)	Unpleasant (b)	Unpleasant (d)	2.90 (0.65)

The interaction between outward appearance and motion was also significant (*F* (1, 15) = 22.3, *p* < 0.0001, *η_p_*^2^ = 0.598). Post-hoc analyses using the Holm-Bonferroni method for the simple main effects revealed the following results: First, regarding the effect of motion, when the outward appearance was pleasant (Figure 3, left, Conditions A vs. B), characters with pleasant motion were evaluated as significantly more pleasant than those with unpleasant motion (*p_adj_* < 0.0001). Similarly, when the outward appearance was unpleasant (Figure 3, left, Conditions C vs. D), characters with a pleasant motion were evaluated as significantly more pleasant than those with an unpleasant motion (*p_adj_* = 0.0159). Second, regarding the effect of outward appearance, when the motion was pleasant (Figure 3, left, Conditions A vs. C), characters with pleasant outward appearance were evaluated as significantly more pleasant than those with unpleasant outward appearance (*p_adj_* < 0.0001). Similarly, when the motion was unpleasant (Figure 3, left, Conditions B vs. D), characters with a pleasant outward appearance were evaluated as significantly more pleasant than those with an unpleasant outward appearance (*p_adj_* = 0.0174).

Taken together, these results suggest that both outward appearance and motion strongly influence the pleasantness score. In addition, while each factor generally enhanced positive feelings independently, the significant interaction indicated that the highest positive evaluations occurred synergistically when a pleasant appearance was paired with a pleasant motion.

#### 3.1.1 Unpleasantness evaluation scores in the main study

We next examined how negative feelings were evoked by outward appearance and motion in the main study (Figure 3, right). The main effect of outward appearance was significant, indicating that an unpleasant appearance (Conditions C and D) evoked significantly more negative feelings than a pleasant appearance (Conditions A and B) (*F* (1, 15) = 55.6, *p* < 0.0001, *η_p_*^2^ = 0.788). Similarly, we found a significant main effect of motion, with unpleasant motion (Conditions B and D) evoking more negative feelings than pleasant motion (Conditions A and C) (*F* (1, 15) = 15.9, *p* = 0.0012, *η_p_*^2^ = 0.514). These observations were consistent with the evaluation scores in the pilot study, which examined different participants (Tables 3–5; see the last section for more details).

The interaction between outward appearance and motion was also significant (*F* (1, 15) = 7.96, *p* = 0.013, *η_p_*^2^ = 0.347). Post-hoc analyses using the Holm-Bonferroni method for the simple main effects revealed the following results: First, regarding the effect of motion, when the outward appearance was pleasant (Figure 3, right, Conditions A vs. B), characters with unpleasant motion were evaluated as significantly more unpleasant than those with pleasant motion (*p_adj_* = 0.001). In contrast, when the outward appearance was unpleasant (Figure 3, right, Conditions C vs. D), there was no significant difference between pleasant motion and unpleasant motion (*p_adj_* = 0.134). Second, regarding the effect of outward appearance, when the motion was pleasant (Figure 3, right, Conditions A vs. C), characters with unpleasant outward appearance were evaluated as significantly more unpleasant than those with pleasant outward appearance (*p_adj_* < 0.0001). Similarly, when the motion was unpleasant (Figure 3, right, Conditions B vs. D), characters with unpleasant outward appearance were evaluated as significantly more unpleasant than those with pleasant outward appearance (*p_adj_* = 0.002).

Taken together, these results suggest that the effect of outward appearance on the unpleasantness score was larger than that of motion. In addition, while both factors generally enhanced negative feelings, an unpleasant appearance led to high unpleasantness regardless of motion. Furthermore, the significant interaction indicated that the effect of unpleasant motion on negative feelings was observed when the outward appearance was pleasant, but not when the outward appearance was unpleasant.

#### 3.1.2 Comparison of evaluation score between the pilot and the main study

Lastly, we examined the consistency of the evaluation scores between the pilot and the main studies, which were obtained from different participants. To take care of the differences in the experimental design (e.g., the presence of neutral stimuli in the pilot study), we calculated the average scores of the relevant paired stimuli from the pilot study (Table 5) and compared them with the corresponding scores from the main study (Table 3). For example, to calculate the average pleasantness score for the combination of pleasant outward appearance and unpleasant motion (Table 5, Condition B), we averaged the scores of conditions (a) and (d) from the pilot study (Table 4). Two-tailed Welch’s t-tests were performed separately for pleasantness and unpleasantness scores across the four stimulus combinations.

The results revealed no significant differences between the two studies in any of the conditions: First, for the combination of pleasant outward appearance and pleasant motion (Tables 3, 5, see Condition A), neither pleasantness (*t* (27) = −0.158, *p* = 0.875) nor unpleasantness scores (*t* (27) = 0.780, *p* = 0.442) showed significant differences. Second, for the combination of pleasant outward appearance and unpleasant motion (Tables 3, 5, see Condition B), there were no significant differences in pleasantness (*t* (27) = −1.892, *p* = 0.070) or unpleasantness scores (*t* (27) = 1.48, *p* = 0.152). Third, for the combination of unpleasant outward appearance and pleasant motion (Tables 3, 5, see Condition C), no significant differences were found in pleasantness (*t* (27) = −1.179, *p* = 0.249) or unpleasantness scores (*t* (27) = −1.65, *p* = 0.111). Fourth and finally, for the combination of unpleasant outward appearance and unpleasant motion (Tables 3, 5, see Condition D), pleasantness (*t* (27) = 0.725, *p* = 0.475) and unpleasantness scores (*t* (27) = 0.197, *p* = 0.845) also showed no significant differences. These results suggested that evaluation scores in the pilot and main study were consistent across different participants.

In summary, outward appearance and motion significantly influence emotional evaluations in both positive and negative scores. For pleasantness, these factors work synergistically to enhance positive feelings. Conversely, for unpleasantness, an unpleasant appearance acts as a dominant factor, masking the influence of motion, indicating that an unpleasant appearance inherently triggers negative feelings regardless of the character’s movement.

## 4 Discussion

### 4.1 Mixed affective responses associated with appearance-motion incongruency

In this study, we defined “kimo-kawaii” as a mixed feeling consisting of pleasant and unpleasant emotions and examined whether 3D characters with incongruent factors evoked mixed feelings such as “kimo-kawaii.” The results of pleasant and unpleasant evaluation scores in the main study showed different effects of outward appearances and motions on emotional evaluations between two categories of incongruent 3D characters. Pleasant evaluation scores (Figure 3, left) showed an interaction between the outward appearances and the motions as follows. For the 3D characters of pleasant outward appearance and unpleasant motion (B in Figure 3, left), these incongruent characters evoked less pleasant emotion than pleasant congruent characters, and more pleasant emotion than unpleasant congruent characters. For the 3D characters of unpleasant outward appearance and pleasant motion (C in Figure 3, left), these incongruent characters also evoked less pleasant emotion than pleasant congruent characters, and more pleasant emotion than unpleasant congruent characters. These two results revealed that the pleasantness of both incongruent characters appeared between the two emotionally congruent characters.

Unpleasant evaluation scores (Figure 3, right) showed the interaction between the outward appearances and the motions as follows. For the 3D characters of pleasant outward appearance and unpleasant motion (B in Figure 3, right), these incongruent characters evoked more unpleasant emotion than pleasant congruent characters, and less unpleasant emotion than unpleasant congruent characters. However, for the 3D characters of unpleasant outward appearance and pleasant motion (C in Figure 3, right), these incongruent characters evoked unpleasant emotions, similar to unpleasant congruent characters. These two results revealed that the unpleasantness of only one incongruent character (i.e., pleasant outward appearance and unpleasant motion) appeared between both emotionally congruent characters. On the other hand, the unpleasantness of other incongruent characters (i.e., unpleasant outward appearance and pleasant motion) was similar to that of unpleasant congruent characters. This suggests that the unpleasant outward appearance strongly evoked unpleasant emotions, effectively overriding the influence of the pleasant motion.

Summarizing the results of pleasant and unpleasant evaluation scores, the emotional effect of 3D characters of pleasant outward appearance and unpleasant motion were observed between the two congruent characters. Thus, this pattern was associated with simultaneous positive and negative affective responses and may be relevant to mixed affective experiences such as “kimo-kawaii.”

### 4.2 Effect of outward appearance and motion on the evaluation scores during the main study

The results of the comparison between the evaluation scores between the pilot and the main studies showed a descriptive pattern for the incongruent stimuli. In both incongruent stimuli, the characters were unpleasant or not pleasant compared to the individually presented (i.e., presentation with neutral condition). However, none of these differences reached statistical significance. On the other hand, in the congruent stimuli, there are no differences between the pilot and the main studies. Therefore, it was suggested that the change in the impression of the character was descriptively observed in the incongruent combination. It might be possible that this change in impression could constitute the “kimo-kawaii” emotions.

Our results suggest that the combination of pleasant outward appearance and unpleasant motion was associated with simultaneous positive and negative affective responses in the present 3D character stimuli. This affective pattern may be relevant to the experience of “kimo-kawaii”; however, because the present study did not use a construct-specific measure of “kimo-kawaii,” this interpretation should be considered exploratory. In contrast, the combination of unpleasant outward appearance and pleasant motion evoked unpleasant emotions as strongly as the combination of unpleasant outward appearance and unpleasant motion. This observation implies that unpleasant outward appearance more strongly influences the participants than pleasant outward appearance. Therefore, the combination of unpleasant outward appearance and pleasant motion would be regarded only as unpleasant because the unpleasantness of the outward appearance strongly influences the participants.

Another interpretation of these results is that the effect of unpleasant motion would have been limited in these stimuli. The motion stimulus we chose (No. 18 in Table 2) was evaluated as less unpleasant than the other stimuli in the pilot study, while differences in the pleasantness and unpleasantness scores in other stimuli were one point or greater when compared to this stimulus (No. 18 in Table 2). Therefore, the combination of unpleasant outward appearance and unpleasant motion would be evaluated as more unpleasant than that of unpleasant outward appearance and pleasant motion when combined with a more unpleasant motion. In future studies, more strongly pleasant or unpleasant stimuli are recommended to be used to examine this hypothesis.

### 4.3 Theoretical implications for mixed emotion and visual affective perception

Our findings contribute to the broader theoretical frameworks of bivariate affect representation and visual affective perception. Traditional circumplex models conceptualize emotional valence along a bipolar continuum (Russell, 1978), whereas bivariate models allow positive and negative affect to be co-activated by complex or incongruent stimuli (Larsen et al., 2001). In a comprehensive meta-analysis of mixed-emotion research, Berrios et al. (Berrios et al., 2015) demonstrated that the elicitation and measured magnitude of mixed emotions vary depending on the measurement paradigm and characteristics of the eliciting stimuli. Our findings extend this perspective by demonstrating that structural incongruity between static appearance and dynamic motion serves as a specific visual condition under which positive and negative affect can be co-activated.

Furthermore, our finding that outward appearance exerted a stronger influence over motion provides a potential perspective on how different visual features contribute to affective evaluation of virtual characters. Previous work has shown that inadequate facial motion in virtual characters can increase perceived uncanniness, with the magnitude of this effect varying across emotions (Tinwell et al., 2011). Another possible interpretation is that incongruent motion may make the character’s emotional state, intention, or behavioral pattern more difficult to interpret or predict (Numata et al., 2024). Such ambiguity in the perception of emotional or behavioral information may contribute to an eerie or unsettling impression (Tinwell et al., 2011). However, the present study did not directly measure perceived intention, emotional-state ambiguity, or behavioral predictability. Therefore, these factors represent possible alternative explanations that should be examined in future studies. Complementing this psychological evidence, recent computational approaches to visual aesthetic evaluation have explicitly integrated emotional and aesthetic features, highlighting the close relationship between emotional information and visual evaluation (Lan et al., 2024; Cheng et al., 2024). From this perspective, our findings suggest that static appearance may establish a relatively strong affective impression, whereas incongruent motion can modulate rather than completely override this initial evaluation. Thus, mixed affect may emerge from the interaction between a strong appearance-based affective impression and conflicting dynamic information.

## 5 Limitations and future directions

Several limitations of the present study should be noted. First, regarding statistical power and generalizability, the sample size in the main study was relatively small ( N=16 ), consisting of university students. A sensitivity power analysis indicated that the present sample size had limited sensitivity to small-to-moderate effects, although it had 80% power to detect effects of d_z_ ≥ 0.75. Although the observed effects were large in magnitude ( $\eta_{p}^{2}$ = 0.347–0.834), these effect-size estimates should be interpreted cautiously given the small sample size, and replication with larger and more demographically diverse samples is warranted.

Second, regarding measurement validity, while we defined “kimo-kawaii” as a mix of pleasant and unpleasant emotions and employed items from a general multiple mood scale, our findings directly demonstrate the co-activation of positive and negative affect rather than the domain-specific construct of “kimo-kawaii” per se. The specific experience of “kimo-kawaii” is often characterized by more nuanced dimensions, such as a combination of “cute” (kawaii) and “eerie” or “disgusting.” Although using a generalized mood scale enabled us to capture broad affective co-activation elicited by visual incongruity, future research should incorporate construct-specific validation scales targeting these precise emotional attributes. The pleasantness and unpleasantness indices were constructed by selecting and averaging items from the original multiple mood scale (Terasaki et al., 1992). However, this specific item grouping has not been independently validated as a measurement scale, and its internal consistency was not separately examined in the present study. Thus, the present indices should be interpreted as measures of broad positive and negative affect rather than as fully validated measures of specific emotional constructs.

Third, the present study adopted an extreme-stimulus selection strategy by choosing the most distinctly evaluated appearances and motions from the pilot study. While this proof-of-concept design effectively maximized affective contrast to examine the interaction between valence components, it may have amplified the observed effect sizes. Furthermore, appearance and motion stimuli may vary along dimensions other than valence, such as arousal, visual complexity, anthropomorphism, familiarity, and motion speed. Although CG character models and motion exposure durations were standardized, these unmeasured perceptual dimensions could have interacted with emotional evaluations. Future studies should quantify and control for these potential confounds across a broader, more continuous spectrum of stimuli to verify the generalizability of these findings. In addition, because the present study focused on human-like 3D characters, it remains unclear whether similar affective responses would be observed for animals, robots, mascots, or non-living objects. Future studies should therefore examine whether the observed relationship between appearance and motion generalizes across different categories of visual stimuli.

## 6 Conclusion

In summary, the present study demonstrated that, under specific conditions, combining a pleasant outward appearance with unpleasant motion in 3D character stimuli can elicit both pleasant and unpleasant affective responses. In contrast, the combination of an unpleasant outward appearance and pleasant motion primarily elicited unpleasant affect. These findings suggest that, within the present stimulus set, outward appearance may exert a stronger influence on affective evaluation than motion. However, because the present study used a limited set of human-like 3D characters and generalized affective measures, further research is needed to determine whether these findings extend to other types of stimuli and to the domain-specific experience of “kimo-kawaii.”

## Data Availability

The raw data supporting the conclusions of this article will be made available by the authors, without undue reservation.
