# Supplementary material for: Mixed emotions evoked when incongruent outward appearance and motion are combined
Source: Front Neurosci. 2026 Oct 6;20:1976417. doi: 10.3389/fnins.2026.1976417 (PMC13680470; doi:10.3389/fnins.2026.1976417)
Supplement: Supplementary file 1 [file Table_1.DOCX]

**APPENDIX**

**Supplementary Table 1 URLs for all stimuli presented in the pilot study**

| (a) Pleasant outward appearance | |
| --- | --- |
| No. 1 | https://sites.google.com/site/kanseienshu2014/mitame01 |
| No. 2 | https://sites.google.com/site/kanseienshu2014/mitame02 |
| No. 3 | https://sites.google.com/site/kanseienshu2014/mitame03 |
| No. 4 | https://sites.google.com/site/kanseienshu2014/mitame04 |
| No. 5 | https://sites.google.com/site/kanseienshu2014/mitame05 |
| (b) Unpleasant outward appearance | |
| No. 6 | https://sites.google.com/site/kanseienshu2014/mitame06 |
| No. 7 | https://sites.google.com/site/kanseienshu2014/mitame07 |
| No. 8 | https://sites.google.com/site/kanseienshu2014/mitame08 |
| No. 9 | https://sites.google.com/site/kanseienshu2014/mitame09 |
| No. 10 | https://sites.google.com/site/kanseienshu2014/mitame10 |
|  |  |
| (c) Pleasant motion | |
| No. 11 | https://sites.google.com/site/kanseienshu2014/ugoki01 |
| No. 12 | https://sites.google.com/site/kanseienshu2014/ugoki02 |
| No. 13 | https://sites.google.com/site/kanseienshu2014/ugoki03 |
| No. 14 | https://sites.google.com/site/kanseienshu2014/ugoki04 |
| No. 15 | https://sites.google.com/site/kanseienshu2014/ugoki05 |
| (d) Unpleasant motion | |
| No. 16 | https://sites.google.com/site/kanseienshu2014/ugoki06 |
| No. 17 | https://sites.google.com/site/kanseienshu2014/ugoki07 |
| No. 18 | https://sites.google.com/site/kanseienshu2014/ugoki08 |
| No. 19 | https://sites.google.com/site/kanseienshu2014/ugoki09 |
| No. 20 | https://sites.google.com/site/kanseienshu2014/ugoki10 |
