# Supplementary material for: Mixed emotions evoked when incongruent outward appearance and motion are combined
Source: Front Neurosci. 2026 Oct 6;20:1976417. doi: 10.3389/fnins.2026.1976417 (PMC13680470; doi:10.3389/fnins.2026.1976417)
Supplement: Supplementary file 2 [file Table_2.DOCX]

**APPENDIX**

**Supplementary Table 2 URLs for all stimuli presented in the main study**

| (A) Pleasant outward appearance - pleasant motion | |
| --- | --- |
| No. 1 | https://sites.google.com/site/kanseienshu201402/movie01 |
| No. 2 | https://sites.google.com/site/kanseienshu201402/movie02 |
| No. 3 | https://sites.google.com/site/kanseienshu201402/movie05 |
| No. 4 | https://sites.google.com/site/kanseienshu201402/movie06 |
| (B) Pleasant outward appearance - unpleasant motion | |
| No. 5 | https://sites.google.com/site/kanseienshu201402/movie03 |
| No. 6 | https://sites.google.com/site/kanseienshu201402/movie04 |
| No. 7 | https://sites.google.com/site/kanseienshu201402/movie07 |
| No. 8 | https://sites.google.com/site/kanseienshu201402/movie08 |
| (C) Unpleasant outward appearance - pleasant motion | |
| No. 9 | https://sites.google.com/site/kanseienshu201402/movie09 |
| No. 10 | https://sites.google.com/site/kanseienshu201402/movie10 |
| No. 11 | https://sites.google.com/site/kanseienshu201402/movie13 |
| No. 12 | https://sites.google.com/site/kanseienshu201402/movie14 |
| (D) Unpleasant outward appearance - unpleasant motion | |
| No. 13 | https://sites.google.com/site/kanseienshu201402/movie11 |
| No. 14 | https://sites.google.com/site/kanseienshu201402/movie12 |
| No. 15 | https://sites.google.com/site/kanseienshu201402/movie15 |
| No. 16 | https://sites.google.com/site/kanseienshu201402/movie16 |
